# Dairy Animal Ownership and Household Milk Production Associated with Better Child and Family Diet in Rural Nepal during the COVID-19 Pandemic

**DOI:** 10.3390/nu14102074

**Published:** 2022-05-16

**Authors:** Laurie C. Miller, Sumanta Neupane, Neena Joshi, Mahendra Lohani, Keshav Sah, Bhola Shrestha

**Affiliations:** 1 Friedman School of Nutrition Science and Policy, Tufts University, Boston, MA 02111, USA; 2 Neupane: Poverty, Health, and Nutrition Division, International Food Policy Research Institute, New Delhi 110012, India; Sumanta.neupane@cgiar.org; 3 Heifer International, Little Rock, AR 72202, USA; Neena.joshi@heifer.org (N.J.); Mahendra.lohani@heifer.org (M.L.); 4 Heifer Nepal, Kathmandu 44700, Nepal; Keshav.sah@heifer.org (K.S.); Bhola.shrestha@heifer.org (B.S.)

**Keywords:** child nutrition, family nutrition, household milk production, dairy animal, diet quality

## Abstract

The economic and health crises related to the COVID-19 pandemic raised considerable concern about child and family diet, especially among small-holder farming households in low- and middle-income countries (LMIC). In rural Nepal, 309 families (including 368 children aged 6–66 months) were enrolled pre-COVID-19 in a prospective study of a nutrition education intervention and family milk consumption. The intervention could not be implemented due to COVID-19; however, child and family diet was assessed in three household surveys (one before and two during the pandemic). Over time, after adjusting for child and household factors, child and family diet quality declined (reduced diet diversity, consumption of milk and animal-source-foods (ASF)). However, in dairy-animal-owning (vs. non-dairy-animal-owning) households, both children and family were more likely to consume milk (aOR respectively 2.88× (*p* < 0.05), 5.81× (*p* < 0.001)). Similarly, in households producing >3.5 L/d milk (vs. ≤3.5 L/d), children and family members were more likely to consume milk (respectively 7.45× and 11.88× (both *p* < 0.001)). Thus, the overall decline in child and family diet quality, especially related to milk consumption, was buffered independently by household ownership of ≥1 dairy animals (cow or buffalo) and by milk production >3.5 L/day. A better understanding of these protective factors might facilitate the development of interventions to promote resilience in future crises.

## 1. Introduction

Over the past 3± years, the world has grappled with the COVID-19 pandemic. Beyond the tragic deaths of nearly 6 million people [1], the economic and personal costs of the pandemic have been profound, affecting education, food security, and employment [2]. This burden has been borne disproportionately by low-income individuals, especially in low- and middle-income countries (LMIC) [3,4]. These individuals and their households have few buffering resources and limited resilience. In Nepal, the pandemic touched every area of this developing and largely agrarian economy, which contracted by ~2% in 2019–2020 (compared to 2018–2019) and was characterized as “shattered” [5]. For example, a loss of income was reported among 85% of rural Nepalese households interviewed in June and mid-July 2020, and was even higher in remote areas [6]. The restrictions adopted by the government of Nepal to control the spread of the virus created a unique “income shock” which gave rise to unprecedented challenges in terms of achieving the United Nations SDGs 1 (no poverty) and 2 (zero hunger) [7].

Rural small-holder farmers were unduly affected by these restrictions. The agricultural supply chain for the sale, storage and supply of perishable agriculture commodities like vegetables, poultry products and dairy products was severely compromised. Lower demand for farm products (due to school, hotel and restaurant closures, event cancellations and restrictions on tourism) combined with disruptions and/or price increases in the input supply chain (e.g., fertilizer, seeds), reduced availability of workers (due to travel restrictions) and livestock market closures all contributed to this burden [6,7,8,9]. In addition, in the early days of the pandemic, there was a widespread belief that the virus was transmitted via foods such as meat, milk, fish and others, further reducing demand for these products [7]. The severity of the economic crisis in the early lockdowns was so severe that some rural populations were more fearful of dying of hunger than of COVID-19 [10].

In parallel with concerns about farming families, considerable alarm was raised about the potential impact of the COVID-19 pandemic on children. Although largely resistant to the serious health risks of COVID-19, children in low-resource rural households were expected to be particularly affected by secondary effects of the pandemic. The predicted rise in poverty levels and reduction in household economic growth and resources were expected to increase household food insecurity, negatively affecting child diet, growth, and development. Experts predicted an alarming increase in child undernutrition in developing countries due to steep declines in household incomes, changes in the availability and affordability of nutritious foods, and interruptions to health, nutrition and social protection services [6,11,12,13].

The story of how COVID-19 impacted the nutritional status of children in LMIC small-holder farms is still unfolding. In the early days of the pandemic, some experts contended that smallholder farming systems would be less resilient to shocks, having fewer available support structures (e.g., lack of adequate storage facilities and infrastructure) to decrease the impact and increase the rate of recovery after the shock [6,8]. Others predicted that small-holder families would be protected to some extent. For example, a 2021 systematic review found that shorter value chains (typical of small-holder farmers) promoted resilience in the face of COVID-19-mandated movement restriction measures [6]. Additional protection from economic shocks was expected to result from the ability of small-holder farmers to rely on subsistence farming [10,14] and direct access to livestock.

Livestock ownership has been said to provide relative resistance to external shocks such as droughts and floods [15,16], and to contribute to household food security [17,18,19]. Livestock ownership has also been associated with better child nutritional status, probably via increased child consumption of animal source foods (ASF) (widely considered to relate to better child nutrition [20,21,22,23,24,25,26,27,28,29,30,31]). Possible pathways include the use of increased income from sales of livestock products to purchase better quality diet items and/or increased availability of home-produced ASF for consumption [32]. However, although many small-holder households keep livestock, ASF consumption by the children is not assured. The relationship between livestock ownership and child nutrition is “not straightforward” [16,21,29,30,32,33]. The relation of livestock ownership to child nutrition during the pandemic has not been fully characterized. Animal milk is of particular interest as it provides micro- and macronutrients which are needed for good nutrition, including calcium, riboflavin, folate, vitamins A and B-12, high-quality protein, whey, casein, linoleic and alpha-linoleic acid [22,34,35]. In developing countries, milk is generally one of the largest sources of animal-based dietary protein [36], and has thus been a special focus of research [20,21,22,28,31,34,35,36,37,38,39,40]. Milk, along with other ASF, also offers important nutrients to adolescents and adults [41,42,43,44,45,46,47,48,49,50]. 

We had the unplanned opportunity to prospectively assess child and family diet in relationship to dairy animal ownership during the pandemic in rural Nepal. The pandemic emerged shortly after we obtained the baseline survey for a planned nutrition intervention project. The project was originally designed to assess the impact of a mothers’ group-based educational intervention on child and family diet, focusing in particular on milk consumption in relation to household dairy animal ownership. Unfortunately, the intervention could not be implemented as planned due to government-imposed restrictions on travel, household visits and group meetings. In the face of these difficulties, we reformulated our research question and hypothesized that diet would worsen over the course of the pandemic as the economic situation of families declined, but that dairy animal ownership would provide some buffering measures to protect child and family diet quality. We were able to conduct two follow-up household surveys to assess child and family diet in 2020–2021. We collected multiple measures of child milk consumption, along with other child and family dietary measures, and were able to relate these to several facets of household dairy animal ownership during the pandemic. The analysis of these three rounds of surveys is the subject of this report. 

## 2. Methods

### 2.1. Ethics

This investigation was approved by the Nepal Health Research Council, as well as the Human Investigation Review Board of Tufts University, and was registered at ClinicalTrials.gov (NCT03886467). Informed consent was obtained from all subjects involved in the study. The consent process was conducted in accordance with the regulations of these two review boards. Two types of consent were available, i.e., for literate and for non-literate individuals. Literate individuals were provided with written materials, which were reviewed with them by one of the field enumerators. For non-literate individuals, the consent form was read aloud by the field enumerator. In both cases, enumerators responded to any questions from the respondents. Consent forms were signed by the literate participants; oral consent was provided by the non-literate individuals. In both cases, consent was witnessed by another of the field enumerators. Consent was obtained prior to commencing each household visit. 

### 2.2. Study Design

Study of child and family diet was the focus of a longitudinal controlled impact evaluation trial [51] implemented by Heifer International Nepal in Bardiya and Dang districts in western Nepal. This area in the Terai (plains region) is largely populated by low-income subsistence farmers. Heifer International Nepal is a non-governmental organization concerned with poverty alleviation via livestock management practices and community empowerment. Heifer’s field activities are provided to specific areas at the request of local non-governmental organizations (NGOs) formed within rural communities. The study was developed as a follow-up to prior work in these districts conducted by Heifer which focused on optimizing household dairy animal husbandry practices and milk production, as well as a separate investigation in nearby districts which assessed the relation of training in family nutrition (with and without concurrent community mobilization activities) on child growth and diet. 

For the purposes of this study, 46 villages and settlements in three municipalities in these districts were matched according to their sociodemographic characteristics including geographic location, altitude, population size, local natural resources, employment opportunities, availability of health care, type of agriculture practiced and prevalence of dairy animal (cow and buffalo) ownership. Four non-adjacent groups of villages within this zone were identified; two were randomly assigned to “Intervention” and two to “Control” status. It was planned for the intervention communities to receive intensive nutrition training during the study period, and that three household visits would be conducted over the intended project period of 18 months. However, due to the pandemic, nutrition training could not be implemented as planned (see below). 

### 2.3. Field Procedures

A professional research organization, with no connection to Heifer was engaged to conduct the household visits. The organization’s field enumerators received 7 days’ didactic and practical training before each survey round. Households were invited to participate if they had at least one child aged 6–66 months. For each household visit, field enumerators completed a 152-item questionnaire with the child’s mother; a supervisor was also present for part of each visit. The core of the questionnaire was based on the Nepal Demographic and Health Survey [52] (household demographics, child and household diet information) with additional modules to assess relevant indicators as described below. 

### 2.4. Impact of COVID-19 on Field Activities

**Figure 1:**
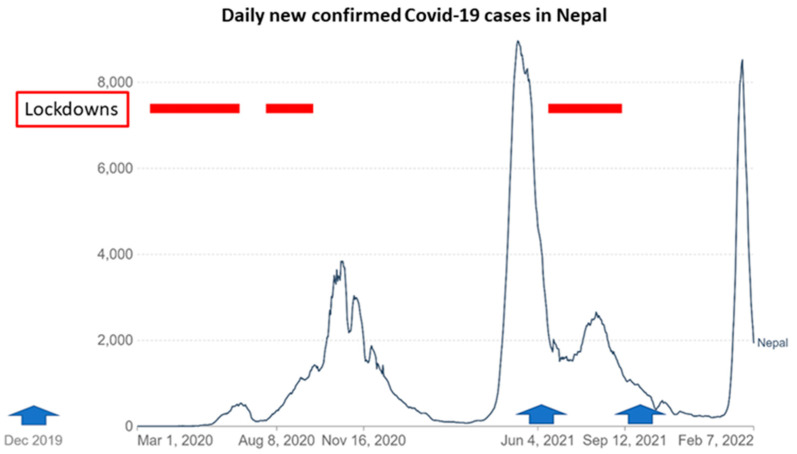
Daily new confirmed COVID-19 cases in Nepal March 2020–2022. Red bars indicate national lockdowns. Blue arrows indicate the three rounds of data collection. The second round survey was conducted in part via telephone. Graph is modified from Data source: Johns Hopkins University CSSE COVID-19 Data. Graphic prepared by Our World in Data (open access) [53].

The baseline assessment was conducted in December 2019. Unfortunately, the COVID-19 pandemic disrupted nearly all project activities. Very limited nutrition training could be provided (5 days only out of 12 planned months). The second and third surveys were postponed until July 2021 and September 2021, respectively, due to the unsafe sanitary situation (Figure 1). (These could not be further spaced out due to financial and logistical constraints.) In addition, the second survey was interrupted by the unexpected urgent re-imposition of government restrictions after 147 households had been visited; information for the remaining 125 households (46%) was collected by telephone interview only (child anthropometry could therefore not be obtained in these households). Although the intended aims of the project were not achieved, information about changes in child and family diet during the pandemic was collected. 

### 2.5. Participants

Households in rural Nepal typically consist of several related adult couples and their children. In this study, 309 mothers and fathers in 251 households and their 368 children (213M:155F) between the ages of 6–66 months were enrolled. Child age was determined by inspection of the birth or vaccine certificate. Children with physical or neurologic handicaps that prevented the consumption of a normal diet for their age or children with severe inter-current illnesses at the time of survey were excluded. Two hundred and fifty-four children were evaluated in all three surveys, 38 in two surveys, and 76 in only one survey. Given the long interval between Round 1 and Round 3, many of the children enrolled at baseline were >66 months of age by Round 3. Accordingly, in the final round, 40 younger children in the same households were enrolled in order to increase the sample size for analysis. (These children had been too young to enroll at baseline). 

## 3. Diet

In each round, respondents were asked if each enrolled child in the household had consumed any of 12 specific foods/food groups within the past 24 h [54]. The number of times the child had consumed each item during the previous week was also recorded. We also asked if “anyone else in the household” (not including the enrolled child) had consumed each of these items within the past 24 h. This is designated throughout as “family diet”. Diet intake questions used local terminology for common foods and included detail to capture all items in each category (for example, the question regarding grain intake was “Did your child consume rice, chapattis or other foods made from millet, maize or lito?”.

The 12 foods/food groups were aggregated into seven categories, coded as “yes” or “no” in each survey round: (1) milk and other dairy products; (2) meat, offal, fish; (3) eggs; (4) starchy staples (grains and white potatoes); (5) vitamin-A rich fruits and vegetables including leafy greens; (6) other fruits and vegetables; and (7) legumes, nuts, and seeds [55,56]. This information was used to construct four key dietary indicators for the enrolled child in each round: (1) A dietary diversity score (DDS, range 0–7), based on the number of categories consumed; (2) the number of ASF (meat, fish, offal, eggs, milk, other dairy products; range 0–6) consumed in the previous 24 h; and (3) in the previous week (child only) [46,57]. The 24-h ASF variable was also dichotomized as (4) “no ASF” or “at least one ASF” consumed. Three parallel family diet indicators were constructed, based on consumption within the prior 24 h: (1) A DDS, (range 0–7), based on the number of food categories consumed; (2) the number of ASF (meat, fish, offal, eggs, milk, other dairy products; range 0–6) consumed; and (3) “no ASF” or “at least one ASF” consumed. For some analyses, ASF consumption was disaggregated into three specific categories: milk (including other dairy), meat (including fish, offal), and eggs. 

In addition, as there was special interest in dairy consumption, six milk-specific indicators were assessed for children. Yogurt was included in several of these variables, as it is widely consumed in these areas (cheese is almost never consumed). Four of the milk-specific indicators related to intake on the previous 24 h: (1) whether or not the child consumed any milk or yogurt; (2) the volume of fresh milk in ml consumed by the child; (3) the adequacy of this volume given the child’s age (for children 7–12 months, >200 mL/day, and for older children, >500 mL/day) [58,59]; and (4) the amount of milk consumed per mL/kg body weight; (children were weighed using standard techniques at each survey round). Two additional milk-specific indicators reflected consumption during the previous week: (5) the number of times milk or yogurt was consumed; and (6) whether this was ≤7 times. For family diet, we also asked if “anyone else in the household” had consumed milk or yogurt in the prior 24 h. 

Each household was provided with a standard, calibrated measuring cup to serve milk to their children. At the inception of the project, mothers were taught how to read the markers on the cup (50 mL increments from 50–250 mL). Two nutrition Master’s level students from a local dairy technology college also made weekly household visits (between lockdowns) to verify and reinforce mother’s understanding and use of the cup. 

### 3.1. Household Demographic Characteristics

Baseline information also included maternal and paternal educational level, as well as that of the most educated man and woman in each household. The level of education was classified as: (1) none or simple literacy classes; (2) some or completed primary school; (3) some or completed secondary school; or (4) school-leaving certificate or beyond. Demographic information (household wealth, land and animal ownership, annual income) was collected at each survey round from each household. Household wealth scores were derived from a principal component analysis of household possessions and quality of housing (e.g., toilet and water facilities), following DHS-Nepal guidelines [52]. Annual income per household and per capita (NPR, Nepali rupees), and amount of land owned (meters squared) were collected as additional indicators of household status. Household income per capita was used in analyses as family size varied greatly (range 2–17, median 6). Animal ownership was determined by asking respondents in each household how many immature and mature cows, buffalos, sheep, goats, pigs, chickens and other poultry they owned. These results were converted to standardized scores using FAO Global Livestock Units for Asia [60]. The average daily amount of milk (in liters) produced by the household’s cows and buffalo was also recorded, along with the percentages of this amount which were sold and kept for family use. Median milk production per household over the three survey rounds was 3.5 L/d; therefore, for some analyses, households were classified as producing either > or ≤3.5 L/day. 

### 3.2. Statistical Analysis

The sample size of 91 children per group was calculated (for the project as initially conceived) to detect a difference of 10% in milk consumption, with a power of 80% and a two-sided significance level of <0.05. Additional children were enrolled to account for the targeted age range (6–66 months) as well as possible attrition. 

Data were entered and analyzed with JMP 13.1 and Stata, version 15.0. Analyses were conducted at the community, household, and individual levels, starting with a descriptive analysis of the variables, including *t*-tests and analysis of variance with Bonferroni post hoc tests to correct for multiple comparisons, followed by a series of chi-square tests and correlations to assess collinearity. Dependent variables were evaluated with histograms to verify normal distribution. As these data were collected over a three-year period, some results are presented by survey round to provide a picture of the dynamic nature of diet over the observation period. Analyses were conducted at the individual and household levels. Mixed-effect regression models (using the Stata command “xtmixed”) were utilized to study the association between consumption of ASF, milk-specific indicators, DDS or other dietary indicators and survey round as fixed effects, and household clustering as random effects. The model also included additional control variables including household (wealth, income, maternal education, the number of children <15 years of age within the household) and child factors (age, gender). As the intervention could not be implemented as planned and few differences were found between Intervention and Control groups throughout the study period (see Supplementary Table S1), these groups were combined for further analysis (maintaining adjustments for household wealth and income per capita in regressions). 

## 4. Results

### 4.1. Participants

The characteristics of the enrolled households over the three survey rounds are shown in Table 1. More boys than girls were enrolled in the study, i.e., 213 vs. 155. The parents of enrolled children in the present study were relatively well educated, i.e., 44% of mothers and 53% of fathers had obtained their school-leaving certificate (or beyond). Despite the pandemic, household wealth, household income per capita, land and overall livestock ownership did not change significantly during the study period. 

### 4.2. Child Diet

Diet characteristics are shown in Table 2. In Round 1, mean child DDS was 4.5 ± 1.1 in the prior 24 h, and 98% of children had consumed at least 1 ASF. Milk was the most commonly consumed ASF in the past 24 h (97% of children); eggs were consumed by 38% and meat by 26%. During the previous week, children had consumed ASF 16.4 ± 5.9 times; most often, this was milk (12.9 ± 5.1 times/week). 

Significant differences in child diet indicators emerged over the three study rounds (Table 2). Most indicators worsened over time: the number (mean ± SD) of ASF consumed declined (Round 1: 1.7 ± 0.8, Round 2: 1.8 ± 0.9, Round 3: 1.5 ± 0.9, *p* = 0.001). Accordingly, the percentage of children consuming milk, eggs or any ASF in the prior 24 h decreased. The frequency of milk consumption during the previous week declined (Round 1: 12.9 ± 5.1, Round 2: 12.5 ± 6.3, Round 3: 10.9 ± 6.9, *p* = 0.0002), while the % of children who consumed milk ≤7× during the previous week increased substantially (Round 1: 4%, Round 2: 13%, Round 3: 23%, *p* < 0.0001) (the remainder consumed milk eight or more times). Of the six milk-specific variables, three worsened progressively over the three survey rounds while three showed temporary improvement at Round 2, returning to baseline values at Round 3. Surprisingly, the number of times meat was consumed by children in the previous week increased over time (*p* < 0.0001). 

### 4.3. Family Diet

Of the four family diet indicators, there were no significant changes in DDS or milk consumption. The percentage of families consuming ≥1 ASF (not including the enrolled child) remained stable. However, the number of ASF consumed in 24 h increased slightly (Round 1: 1.4 ± 0.8, Round 2: 1.6 ± 0.9, Round 3: 1.6 ± 1.0, *p* = 0.001) over the three survey rounds.

To investigate these relationships in further detail, child and family diet indicators were compared to baseline results after adjustment for survey round, household wealth, household income per capita, number of children <15 years of age in the household, child age and gender, and mothers’ education (Table 3). As expected, some indicators progressively declined. Child DDS was unchanged between Round 1 and Round 2, but diminished at Round 3 (Round 2: β (95%CI) 0.04 (−0.17, 0.25), ns; Round 3: −0.23 (−0.43, −0.02); *p* < 0.05). The adjusted odds that a child consumed ≥1 ASF or milk declined over time (for ASF: 93% lower at Round 2 (*p* < 0.01) and 96% less likely at Round 3 (*p* < 0.001) and for milk: 85% lower at Round 2 and 93% less likely at Round 3 (both *p* < 0.001)). 

In contrast, several diet indicators improved in Round 2, and then returned to baseline values in Round 3. These included the number of ASF consumed by the child in the previous week (Round 2: 2.03 (0.74, 3.32), *p* < 0.01; Round 3: 0.48 (−0.82, 1.77), ns), the 24-h milk volume (Round 2: 50.37 (16.26, 84.48), *p* < 0.01; Round 3: 3.02 (31.13, 37.17), ns), and mL/kg of milk consumed (Round 2: 3.56 (.46, 6.65), *p* < 0.05; Round 3 −0.32 (−2.98, 2.33), ns). The odds that the child consumed an adequate volume of milk was 2.78 times greater in Round 2 compared to Round 1 (aOR 2.78 (1.57, 4.93), *p* < 0.001), but no difference was found when Round 3 was compared to Round 1. Thus, the observed improvement was transient for these indicators. Notably, however, the odds of consuming milk ≤7× in the previous week decreased by 74% in Round 2 and 84% in Round 3 (Round 2: aOR 0.26 (0.14, 0.47), *p* < 0.001; Round 3: aOR 0.16(0.09, 0.28), *p* < 0.001), indicating that children were significantly more likely to have consumed milk >7× in the previous week in both Round 2 and Round 3 compared to baseline. 

Within the family, the total number of ASF consumed increased (Round 2: 0.26 (0.08, 0.43), *p* < 0.01; Round 3: 0.27 (0.10, 0.45), *p* < 0.01), but no changes were seen in DDS or the likelihood of milk consumption. 

### 4.4. Household Dairy Animal Ownership and Milk Production

We next addressed the relationship of child and family diet indicators to household dairy animal ownership and milk production. Both cows and buffalo were kept as dairy animals by these small-holder farmers. Many households kept both dairy cows and buffalo: Round 1—63%; Round 2—74%; and Round 3—60%. In Round 1, 49% of households had at least one dairy cow: (33% had one cow, 8% had two cows, and 8% had three or more cows) and 62% of households had at least one dairy buffalo (46% had one buffalo, 10% had two buffalo, and 6% had three or more buffalo). The number of dairy animals and the quantity of cow and buffalo milk produced, sold and kept in the households are shown in Supplemental Tables S2 and S3. 

### 4.5. Dairy Animal Ownership and Child and Family Diet

We next assessed the relation of dairy animal ownership to diet indicators (Supplementary Table S4). Over the three rounds, dairy animal ownership related to an increasing number of diet indicators. In Round 1, dairy animal ownership was not associated with *any* child diet indicators, and only to one family diet indicator (whether or not someone had consumed milk). In Round 2, more children in households with (vs. without) dairy animals consumed milk (93% vs. 79%, *p* = 0.01) and ≥1 ASF (96% vs. 84%, *p* = 0.009). Likewise, children in households with (vs. without) dairy animals were less likely (9% vs. 24%, *p* = 0.02) to consume milk ≤7 times during the prior week (vs. 8 or more times). Dairy-owning families (vs. non-owners) consumed more ASF (1.73 ± 0.90 vs. 1.23 ± 0.94, *p* = 0.0024) and were more likely to consume ≥1 ASF (95% vs. 76%, *p* = 0.0005) and milk (90% vs. 61%, *p* < 0.0001). 

In Round 3, these relationships between dairy animal ownership and family diet were sustained. All six milk-specific indicators were more favorable for children in dairy-animal-owning households, i.e., they were more likely to have consumed milk (and in larger volumes) in the prior 24 h. Children in dairy-owning households also consumed more ASF in Round 3. Thus, ownership of dairy animals related increasingly to diet quality indicators for both children and family members during the ongoing pandemic. 

### 4.6. Milk Production and Child and Family Diet

We next examined the relationship between the amount of milk produced in each household and the diet indicators for children and family members (Supplementary Table S5). Households were classified based on median milk production over the three survey rounds as producing > or ≤3.5 L/day. Increasing numbers of diet indicators were significantly related to these categories: Round 1—7 indicators; Round 2—12 indicators; and Round 3—13 indicators), suggesting that the quantity of milk produced in the household was more strongly related to measures of diet quality over time. 

We further examined the relationship between dairy animal ownership, milk production and diet indicators after adjusting for study round, household (wealth, income per capita, maternal education, number of children <15 years of age) and child (age and gender) characteristics (Table 4). Dairy animal ownership related strongly to the child milk-specific indicators as well as family ASF and milk consumption. In the prior 24 h, children in dairy-owning-households were 2.88× more likely to have consumed milk (aOR (95% CI) 2.88 (1.29, 6.43), *p* < 0.05), consistently drank more milk (β (95%CI) 58.72 mL (15.44, 102.01, *p* < 0.01)), consumed more milk per kg body weight (β 4.66 mL (0.91, 8.40, *p* < 0.05)) and were 2.31× more likely to have consumed an adequate volume of milk for their age (aOR 2.31 (1.08, 4.94, *p* < 0.05). Children in dairy-owning households consumed milk (β (95%CI) 1.60 (0.28, 2.93, *p* < 0.05)) more times in the prior week. Family diet in dairy-owning households included more ASF (β 35(0.14, 0.55, *p* < 0.01)) and was 5.81× more likely to include milk (aOR 5.81 (3.15, 10.70, *p* < 0.001)). Thus, dairy animal ownership was an important factor in child and family diet intake, especially as related to milk.

Household milk production >3.5 L/d related even more strongly to the diet indicators (Table 4). Children in households producing >3.5 L/d of milk were 7.45× more likely to have consumed milk in the prior 24 h (aOR 7.45 (2.97, 18.69), *p* < 0.001) and 2.12× more likely to have consumed an adequate volume of milk for their age (aOR 2.12 (1.31, 3.43), *p* < 0.01). In households producing >3.5 L/d, children consumed 68 mL/d (*p* < 0.001) and 4.33 mL/kg (*p* < 0.01) more milk than children in household with less milk production. Children in these higher-producing households also consumed 2.21 more ASF and 1.99 more servings of milk per week (both *p* < 0.001). Family diet in the previous day was also better in households producing >3.5 L/d: ASF consumption and DDS were both higher (respectively, +0.24 and +0.31), and families were 11.88× more likely to consume milk (all *p* < 0.001). Thus, milk production volume predicted the diet indicators more strongly than dairy animal ownership.

## 5. Discussion

In addition to serious health risks, the COVID-19 pandemic created enormous economic hardships for many individuals in LMIC. Nepal was particularly hard-hit due to the heavy dependency of its economy on agriculture, tourism and foreign remittance. An increase in food prices was expected to result in increased child malnutrition and mortality [11,12,13,61]. We hypothesized that child and family diet quality would diminish during the pandemic, but that dairy animal ownership would buffer this decline. Prior to the pandemic, child and household diets were similar to what has been previously described in this area of Nepal [27,28,62,63], with low DDS and ASF consumption. Over the 21 months of the pandemic, both child and family diet quality declined. In unadjusted analyses, child DDS and ASF consumption (both at 24 h and 7 days) decreased over time. Progressively fewer children consumed a minimum of 1 ASF in the 24 h prior to each survey. Several measures of milk consumption also declined, i.e., the number of times the child consumed milk in the prior week and the proportion of children who consumed any milk in the prior 24 h; the proportionof children who consumed milk <7× (vs. ≥8×) in past week increased. Family diet quality also worsened, i.e., significantly fewer ASF were consumed over time. However, after adjusting for study round, child and household factors, dairy animal ownership and milk production >3.5 L/d were independently associated with better quality diet, especially related to milk consumption.

Previous research on the determinants of the nutritional status of rural children in LMIC provides the context for these findings. ASF provide high-quality, high-density, and readily bioavailable nutrients, including zinc, vitamins A, B12, iron, and calcium. These critical nutrients are difficult or even impossible to obtain from a plant-based diet without supplements or fortification [22,26,50,64,65]. In developing countries, milk is generally one of the largest sources and most widely consumed forms of ASF [36]. Diet quality—particularly consumption of ASF—is essential for optimal child growth and development [66]. ASF provide important benefits to other vulnerable household members, including pregnant and lactating women, adolescent girls, and elders [41,42,43,44,45,46,47,48], and contribute to diet quality for school children of all ages as well as adult men [47,48,49,50].

Paradoxically, although many small-holder households keep livestock, ASF consumption by the children is not assured [32,33]. This apparent contradiction may relate to intra-household food allocation practices, which often prioritize ASF consumption for the primary household breadwinner (usually an adult male) [67]. Sometimes, limited household ASF production may reduce their availability to children. Alternatively, animal products may be prioritized for sale rather than household consumption. Cultural practices also dictate feeding practices and food preferences [67]. 

Because of the importance of milk, the association between dairy animal ownership and diet has been previously studied [21,29,30,32,33,68]. Cow ownership has been associated with increased dairy consumption in the household in Kenya [68]. Studies in India and Ethiopia show that cow ownership is important for enhancing milk consumption by children and reduces stunting rates [16,36]. In Uganda, families who adopted “improved” cow breeds had higher milk sales and household milk intake [32]. However, in a comprehensive assessment of different measures of livestock ownership in Zambia, no relation was seen between various ownership “typologies” and children’s odds of consuming ASF [33].

Indeed, understanding of the relationship between household dairy ownership and child diet has been characterized variously as “not clear cut” [21], “not straightforward, especially for the small farmers” [32], “not well understood” and “complicated” [29] and “complicated by differing patterns across age groups of children” [16]. The pathways by which livestock ownership relates to child and family nutrition are “complex and context-specific” [33]. Indirect but potentially positive influences on household diet include better access to purified water, sanitation and health care via livestock-associated wealth [29]. Alternatively, empowerment of women and improving crop yields through nutrient cycling, manure fertilizer and draft power may indirectly improve household diet [30,33]. The possible negative contributions of livestock ownership (e.g., EED, zoonotic diseases, increased maternal time burden and energy demands due to the physical labor required to rear livestock, reduced land availability for food crops) to child and family outcomes are also important to consider [29,33].

Interpretation of the relationships between diet and animal ownership is further complicated by the different diet metrics used in research studies. Diet is usually characterized by 24-h food recall, providing a snapshot of recent food intake, including diet diversity and ASF consumption. Milk consumption is usually coded as a “yes/no” variable, offering a very limited view of what the individual actually ingests [69]. Measures of frequency and quantities of milk consumption are rarely included, and if present, are rarely corrected for age or weight. Likewise, metrics to define livestock ownership rarely account for the number of animals, lactation status, milk yield and household use of milk vs. sale. Understanding may be further complicated by the non-random and culturally driven nature of dairy animal ownership [36], which also relates to agro-ecological factors and local infrastructure availability (e.g., availability of food, water, markets, veterinary services) [36]. These factors may be especially important in Nepal, a country characterized by extremely low milk productivity. For example, average annual milk yield per cow in Nepal in was 739 kg, vs. 1550 kg in South Asia (and 2699 globally) in 2019; for buffalo, milk yield was 880 kg in Nepal, vs. 2072 kg in South Asia (and 1993 globally) [70]. As a consequence, Nepal faces a deficit in milk availability, estimated at ~12 kg/year per capita [70]. This situation underlines the importance of household milk production, consumption, and allocation decisions.

Despite these complexities, dairy consumption and dairy animal ownership have been linked to child growth in developed and developing countries [20,22,28,30,31,34,38]. Several studies in East Africa from the 1980–1990s showed a positive relationship between dairy animal ownership and child growth (reviewed in [30]), confirmed more recently in studies linking dairy animal ownership and reduced stunting and/or better height-for-age z scores [16,29,30,35,36,71]. Again, however, these relationships are not straightforward. Some reports rightly emphasize that these relationships are conditional on milk being used for family consumption [16]. Dairy herd size appears to be important: a comprehensive study of DHS data from 3 East African countries found reduced stunting prevalence in Ethiopia and Uganda (but not Kenya) but only with a ten-fold increase in household livestock ownership [29]. The authors thus concluded that there was only a “slightly beneficial effect” of household livestock ownership on child stunting prevalence. In Zambia, no relation was found between livestock ownership and child height-for-age z-score or stunting odds even when multiple measures of livestock ownership were assessed [33].

In this study, we were able to establish a positive association between dairy animal ownership and child and family diets during the ongoing pandemic. Although dairy animal ownership declined during the study period, 81% of families still had at least 1 dairy animal at the final survey round, although the proportion and number of households producing ≤3.5 L/day of milk (combined cow and buffalo) increased during this time. The families who had at least 1 dairy animal or who produced >3.5 L/d had better diet quality. These findings were particularly noteworthy given the pandemic crisis, a time when Nepal’s economy was described as “shattered” [5]. The pandemic presented special challenges to rural small-holder farmers in Nepal, including difficulty maintaining social distancing, costs of sanitizer, masks, COVID-19 testing, etc. [8]. Daily farming activities, i.e., plowing, irrigation, and harvest, did not cease during the pandemic, and these communal activities and the urgency of ensuring food for coming seasons necessarily took precedence over sheltering in place [72]. Restrictions related to the pandemic presented enormous challenges to small-holder farmers, as closed markets reduced the ability of farmers to buy and sell animals and animal products. The benefits of the relatively short supply chain for these farmers were offset by their lack of storage facilities and other infrastructure, and reduced financial and practical ability to withstand market delays and closures [6]. Many farmers had to discard their milk, eggs, and vegetables on the street, as they were unable to cope with the breakdown in market channels [10,73].

Our study has several important limitations. Most importantly, the study design was chosen to assess the impact of a nutrition education intervention. As the intervention could not be implemented as planned, the resulting study was reformulated to take advantage of the longitudinal data collection that occurred during the pandemic. Our relatively small sample size was based on power calculations to address the effects of an intervention and may not have been adequate for the research question in this redesigned study, or for adequately assessing the impact of specific dairy practices on child diet. Additionally, due to the changing mobility restrictions during this difficult period, household visits for the second survey had to be interrupted after 54% of enrolled households had been visited. The remaining 46% were contacted by telephone and responded to the questionnaire orally. We were unable to ascertain if telephone and in-person responses had the same validity. Furthermore, in this subset of 46% of households, we could not obtain weight measurements at Round 2, so were unable to calculate the ml/kg of milk ingested. Overall, milk production decreased over time in these project areas. We were unable to determine if this was due to seasonal factors, reduced feed rations given to the animals (in order to save money and/or to deliberately decrease milk production), decreased number of animals, or other factors [74,75,76,77,78,79]. We were also unable to directly measure the nutritional content of the milk consumed in this study or to differentiate the type of animal milk consumed, as many families that participated in the dietary surveys kept both dairy cows and buffalo. Finally, we were unable to independently verify the amount of milk produced by the dairy animals, and therefore relied on farmers’ reports.

Our study also had several strengths. Despite the need to abandon the planned nutrition intervention, we were able to conduct three household surveys over a period of 21 months before and during the COVID-19 pandemic. The diet information collected allowed us to construct six milk-specific variables to provide a more complete picture of milk consumption than a simple “yes/no” variable describing milk consumption in the prior 24 h. Mothers in the enrolled households were able to accurately report the amount of milk provided to their children, using a standardized measuring cup. We also collected detailed information about family diet. In addition, the number of adult dairy animals and the amount of milk produced, sold, and kept by each dairy-owning household was recorded at each survey round. We were thus able to demonstrate the heterogeneous responses of a variety of dietary indicators, for both young children and their families, over the course of the pandemic, and the importance of dairy animal ownership and milk production to diet quality.

Although the quality of child and family diet generally declined over the course of the pandemic, the presence of adult dairy animals and production of >3.5 L/d of milk in the household were shown to be related to better milk consumption. Our results have several implications for agricultural policies in Nepal. The importance of milk production to child and family diet indicators suggests the importance of dairy animal ownership in terms of “protecting” diet quality. This implies that livestock interventions to increase dairy animal ownership, dairy productivity (including sanitation measures and veterinary care to improve animal health) and dairy market development could conceivably improve family nutrition.

The changing situation of the pandemic over time underscores the complexity of the relationship between the crisis, child and family child diet, and family livestock ownership. In this “real-world” crisis situation in rural Nepal, dairy animal ownership and household milk production were shown to bolster child and family milk consumption and diet quality. Better understanding of the detailed pathways by which this occurred could help prepare farmers and their families for future shocks and other challenges, as well as supporting interventions to promote resilience in the face of crises.

## Figures and Tables

**Table 1:** Child and Household Characteristics. The lower portion of the table shows the changes over the three survey rounds. Only child age changed significantly (shown in bold).

*Child Characteristics*				
Child gender (M:F)	213:155			
Child gender %	58%:42%			
*Parents’ education*				
Mothers’ education (*n* = 309)				
None or basic	32 (10%)			
Some or completed primary school	58 (19%)			
Some or completed secondary school	82 (27%)			
School-leaving certificate or beyond	137 (44%)			
Fathers’ education (*n* = 309)				
None or basic	6 (2%)			
Some or completed primary school	59 (19%)			
Some or completed secondary school	81 (26%)			
School-leaving certificate or beyond	163 (53%)			
*Household and child characteristics over time*	ROUND 1 (*n* = 251)	ROUND 2 (*n* = 230)	ROUND 3 (*n* = 258)	Round 1 vs. Round 2 vs. Round 3
Household				
Wealth score	−0.00 ± 1.00	0.09 ± 0.89	−0.00 ± 1.00	ns
Income per capita (NPR)	65,925 ± 52,299	72,099 ± 55,741	73,240 ± 49,337	ns
Animal score	2.35 ± 2.36	2.51 ± 2.59	2.40 ± 2.49	ns
Land ownership (m^2^)	6272 ± 8198	5941 ± 5214	5884 ± 5171	ns
Number of children <15 years	2.04 ± 1.11	2.12 ± 1.05	2.18 ± 1.06	ns
Child	(*n* = 299)	(*n* = 272)	(*n* = 340)	
Child age (months)	34.3 ± 16.7	51.34 ± 21.1	47.7 ± 16.7	**<0.0001**

NPR = Nepali rupees (100 NPR = 0.83 US cents).

**Table 2:** Child and Family diet all 3 rounds. Diet indicators for children (key and milk-specific) and family are shown over the three survey rounds. Some indicators reflect diet intake over the previous 24 h and others over the previous 7 days. Significant differences (in bold) were found for nearly all child diet indicators over the three survey rounds; only 1 family diet indicator changed significantly over the three survey rounds.

			Round 1 (*n* = 301)	Round 2 (*n* = 272)	Round 3 (*n* = 341)	*p* ^3^
	Time					
Child Key Diet Indicators	24 h	DDS ^1^	4.5 ± 1.1	4.6 ± 1.1	4.3 ± 1.2	***
		Consumed at least 1 ASF ^2^	98%	93%	89%	***
		# of ASF consumed ^1^	1.7 ± 0.8	1.8 ± 0.9	1.5 ± 0.9	***
		Consumed eggs ^2^	38%	32%	22%	***
		Consumed meat ^2^	26%	20%	26%	ns
	7 days	# of times ASF consumed ^1^	16.4 ± 5.9	17.5 ± 8.1	15.3 ± 8.6	**
		# of times eggs consumed ^1^	2.2 ± 2.5	2.3 ± 2.6	1.5 ± 2.3	***
		# of times meat consumed ^1^	0.7 ± 0.8	0.9 ± 0.9	1.2 ± 1.1	***
Child Milk-Specific indicators	24 h	Consumed milk ^2^	97%	89%	79%	***
		Amount of milk consumed (mL) ^1^	298 ± 162	368 ± 204	297 ± 218	***
		Milk volume sufficient for age? ^2^	21%	30%	22%	**
		Milk ml/kg ^1^	24 ± 14	29 ± 16 †	22 ± 17	***
	7 days	# of times milk consumed ^1^	12.9 ± 5.1	12.5 ± 6.3	10.9 ± 6.9	***
		Consumed milk ≤7× ^2^	4%	13%	23%	***
Family ^ Diet Indicators	24 h	DDS ^1^	4.3 ± 1.1	4.5 ± 1.1	4.4 ± 1.1	ns
		# of ASF consumed ^1^	1.4 ± 0.8	1.6 ± 0.9	1.6 ± 1.0	***
		Consumed at least ^1^ ASF ^2^	88%	92%	88%	ns
		Consumed milk ^2^	81%	85%	77%	ns

^1^ mean ± SD; ^2^ dichotomous variable; ^3^ *p* value. DDS—diet diversity score; ASF—animal source food; #—number; ns—not significant. ^ consumed by someone in the household (other than the enrolled child); † only 147 children (of 272 (54%) enrolled in Round 2) had the anthropometry needed for calculating this variable. *** <0.001, ** <0.01.

**Table 3:** Dietary indicators after adjustment for household and child factors. Mixed effect regressions (logistic and linear) were performed to assess the change in diet indicators over the three survey rounds. Analyses were adjusted for survey round, child (age and gender) and household (maternal education, wealth, income per capita, number of children <15 years of age). *** <0.001, ** <0.01, * <0.05; ^1^ Adjusted odds ratio (95%CI); † consumed by someone in the household (other than the enrolled child); DDS dietary diversity score, ASF animal source foods, # number. Bold indicates significant results.

	Time	Indicator	Round 2	Round 3
Child Key Diet Indicators	24 h	DDS	0.04 (−0.17, 0.25)	−0.227 * **(-0.43, −0.02)**
		# ASF consumed	0.09 (−0.09, 0.26)	−0.08 (−0.26, 0.09)
		Consumed at least 1 ASF ^1^	0.07 ** **(0.01, 0.34)**	0.04 *** **(0.01, 0.18)**
	7 days	# times ASF consumed	2.03 ** **(0.74, 3.32)**	0.48 (−0.82, 1.77)
Child Milk-specific indicators	24 h	Consumed milk ^1^	0.15 *** **(0.05, 0.43)**	0.07 *** **(0.02, 0.19)**
		Amount of milk consumed (mL)	50.37 ** **(16.25, 84.48)**	3.02 (−31.13, 37.17)
		Milk volume sufficient for age? ^1^	2.78*** **(1.57, 4.93)**	1.63 (0.92, 2.88)
		Milk mL/kg	3.56 * **(0.46, 6.65)**	−0.32 (−2.98, 2.33)
	7 days	# times milk consumed	0.19 (−0.86, 1.24)	−0.94 (−1.99, 0.1)
		Consumed milk ≤7× ^1^	0.26 *** **(0.14, 0.47)**	0.16 *** **(0.09, 0.28)**
Family Diet Indicators †	7 days	DDS	0.16 (−0.05, 0.36)	0.04 (−0.17, 0.24)
		# ASF consumed	0.26 ** **(0.08, 0.43)**	0.27 ** **(0.1, 0.45)**
		Consumed at least 1 ASF ^1^	1.32 (0.65, 2.7)	0.89 (0.47, 1.7)
		Consumed milk ^1^	1 (0.54, 1.83)	0.64 (0.36, 1.14)

**Table 4:** Relationship of child and family diet indicators to dairy animal ownership and household milk production. Both dairy animal ownership and household milk production >3.5 L/d were associated with child and family diet indicators in mixed effect regressions (linear and logistic) adjusted for survey round, child (age and gender) and household (maternal education, wealth, income per capita, number of children <15 years of age).

	Time	Indicator	Has at Least 1 Adult Cow or Buffalo	Milk Production >3.5 L/Day
Child Key Diet Indicators	24 h	DDS	−0.02 (−0.26, 0.23)	0.16 (−0.01, 0.33)
		# ASF consumed	0.11 (−0.1, 0.31)	0.14 (0.00, 0.28)
		Consumed at least 1 ASF ^1^	0.89 (0.51, 1.55)	1.12 (0.76, 1.65)
	7 days	# times ASF consumed	1.60 (−0.05, 3.24)	2.21 *** **(1.09, 3.34)**
Child Milk-specific indicators	24 h	Consumed milk ^1^	2.88 * **(1.29, 6.43)**	7.45 *** **(2.97, 18.69)**
		Amount of milk consumed (mL)	58.72 ** **(15.44, 102.01)**	67.98 *** **(38.41, 97.55)**
		Milk volume sufficient for age? ^1^	2.31 * **(1.08, 4.94)**	2.12 ** **(1.31, 3.43)**
		Milk mL/kg	4.66 * **(0.91, 8.40)**	4.33 ** **(1.85, 6.81)**
	7 days	# times milk consumed	1.60 * **(0.28, 2.93)**	1.99 *** **(1.09, 2.9)**
		Consumed milk ≤7× ^1^	1.2 (0.64, 2.27)	1.24 (0.79, 1.96)
Family Diet Indicators †	7 days	DDS	0.2 (−0.03, 0.44)	0.31 *** **(0.15, 0.48)**
		Consumed at least 1 ASF ^1^	4.77 *** **(2.56, 8.89)**	6.116 *** **(3.06, 12.17)**
		# ASF consumed	0.35 ** **(0.14, 0.55)**	0.24 *** **(0.11, 0.37)**
		Consumed milk^1^	5.81 *** **(3.15, 10.7)**	11.88 *** **(6.17, 22.88)**

^1^ adjusted odds ratio (95% CI) *** <0.001, ** <0.01, * <0.05; DDS dietary diversity score, ASF animal source food, # number; † consumed by someone in the household other than the enrolled child. Bold indicates significant results.

## Data Availability

The data and associated information presented in this study are available on request from the corresponding author. The data are not publicly available due to privacy concerns.

## Supplementary Materials

The following supporting information can be downloaded at: https://www.mdpi.com/article/10.3390/nu14102074/s1. Table S1: Household characteristics over the three survey rounds for Intervention and Control groups. Table S2: Household ownership of adult dairy animals. Table S3: Quantities of milk produced, sold and kept by the household. Table S4: Relation of dairy animal ownership and diet indicators. Table S5: Relation between child and family diet indicators and household milk production (combined cow and buffalo). Table S6: Relation of child and family diet indicators to dairy animal ownership. Table S7. Relation of child and family diet indicators to household milk production (> or ≤3.5 L/d).
