# Supplementary material for: Dairy Animal Ownership and Household Milk Production Associated with Better Child and Family Diet in Rural Nepal during the COVID-19 Pandemic
Source: Nutrients. 2022 May 16;14(10):2074. doi: 10.3390/nu14102074 (PMC9147928; doi:10.3390/nu14102074)
Supplement: Supplementary file 1 [file nutrients-14-02074-s001.zip › nutrients-1710340 -Supplementary.pdf]

Table S1. Household characteristics over the three survey rounds for Intervention and Control groups. Wealth scores were higher in Control vs Intervention households in all three rounds, but there were no changes within Intervention or Control groups for any of the four variables shown over the three survey rounds. Significant results are shown in bold.

	Round 1			Round 2			Round 3			Round 1 vs Round 2 vs Round 3	
	Intervention (n = 129)	Control (n = 122)	p	Intervention (n = 120)	Control (n = 110)	p	Intervention (n = 141)	Control (n = 117)	p	Intervention (n = 390)	Control (n = 349)
Wealth	−0.18 ±1.09	0.19 ±0.85	**0.0023**	−0.08 ±1.00	0.29 ±0.72	**0.001**	−0.22 ±1.10	0.27 ±0.78	**<0.0001**	ns	ns
Income per capita	57,986 ±50,213	74,319 ±53,346	**0.01**	68,915 ±62,137	75,573 ±47,843	ns	69,499 ±53,167	77,750 ±44,088	ns	ns	ns
Animal score	2.35 ±2.54	2.35 ±2.16	ns	2.45 ±2.76	2.58 ±2.41	ns	5994 ±4928	5750 ±5467	ns	ns	ns
Land ownership	6204 ±5579	6345 ±10,292	ns	6148 ±5124	5715 ±5324	ns	2.35 ±2.79	2.46 ±2.09	ns	ns	ns

Table S2. Household ownership of adult dairy animals. The number and percentage of households which owned dairy cows or buffalo is shown for each survey round. Households were classified by the number of adult cows or buffalo owned (none, 1, or >1). These proportions did not differ significantly over the three survey rounds. Dairy animal ownership significantly decreased over the study period. Significant results are shown in bold.

	n	Cow Ownership p = ns			Buffalo Ownership p = ns			Any Dairy Animal Ownership (Cow or Buffalo) **χ2 25.74, p < 0.0001**	
		None	1	>1	None	1	>1	None	⩾1
Round 1	251	129 (51%)	83 (33%)	39 (16%)	94 (37%)	116 (46%)	41 (16%)	13 (5%)	238 (95%)
Round 2	230	134 (58%)	57 (25%)	39 (17%)	101 (44%)	95 (41%)	34 (15%)	38 (17%)	192 (83%)
Round 3	258	146 (57%)	69 (27%)	43 (17%)	116 (45%)	101 (39%)	41 (16%)	48 (19%)	210 (81%)

Table S3. Quantities of milk produced, sold and kept by the household. The amounts of cow, buffalo and combined cow plus buffalo milk produced, sold and kept by the household is shown. The combined amount of milk produced, sold and kept decreased over the three survey rounds (as more farmers gave up their dairy animals). HH household. Significant results are shown in bold.

	n	Round 1	*n*	Round 2	*n*	Round 3	*p*
COW							
Produced	112	3.46 ± 9.94	85	3.81 ± 9.03	85	2.33 ± 6.07	ns
Sold	71	5.59 ± 12.99	68	7.82 ± 12.06	58	5.03 ± 8.41	ns
Kept	134	2.15 ± 1.53	78	2.49 ± 1.82	76	2.05 ± 1.65	ns
% of milk produced that was kept	112	56%	85	41%	85	48%	**0.01**
Produced ≤3.5 L/d	45	40%	16	19%	28	33%	**0.006**
#/% of HH where all milk produced kept for family use	41	37%	17	20%	27	32%	**0.03**
#/% of these who produced ≤3.5 L/day	34	83%	10	59%	21	77%	ns
BUFFALO							
Produced	138	2.59 ± 3.32	87	1.94 ± 3.16	71	1.27 ± 2.63	**<0.0001**
Sold	71	2.63 ± 3.03	62	3.02 ± 3.37	53	2.59 ± 2.84	ns
Kept	106	2.10 ± 1.40	82	2.12 ± 1.26	70	2.09 ± 1.59	ns
% of milk produced that was kept	138	55%	87	52%	71	51%	ns
Produced ≤3.5 L/d	54	39%	22	25%	30	42%	ns
#/% of HH where all milk produced kept for family use	43	31%	25	28%	18	25%	ns
#/% of these who produced ≤3.5 L/day	33	76%	14	56%	14	77%	ns
Cow + buffalo milk (L/day)							
Total Prod		6.05 ± 9.91		5.76 ± 9.53		3.61 ± 6.34	**0.0028**
Median		4.5		4		2	
Produced <3.5 L/d	251	41% (103)	230	47% (107)	258	63% (163)	**<0.0001**
Total Sold		3.93 ± 9.31		4.03 ± 8.76		2.36 ± 5.58	**0.032**
Total Kept		2.12 ± 1.55		1.72 ± 1.69		1.25 ± 1.64	**<0.0001**

Table S4. Relation of dairy animal ownership and diet indicators**.** Households were classified as having none or ≥1 adult dairy animal (cow or buffalo). The number of diet indicators related to these categories increased over the three survey rounds. Significant results are shown in bold.

			# of Adult Dairy Animals (Cow or Buffalo)								
	Time	Indicator	Round 1			Round 2			Round 3		
			1 or more	None	*p*	1 or more	None	*p*	1 or more	None	*p*
Child Key diet indicators	24 h	DDS[1]	4.57 ± 1.05	4.61 ± 1.50	ns	4.67 ± 1.01	4.55 ± 1.24	ns	4.36 ± 1.14	4.41 ± 1.25	ns
		# ASF consumed[1]	1.71 ± 0.80	2.07 ± 1.11	ns	1.89 ± 0.90	1.65 ± 1.21	ns	1.68 ± 0.93	1.54 ± 1.14	ns
		Consumed at least[1] ASF[2]	98%	100%	ns	96%	84%	**0.009**	91%	84%	ns
	7 days	# times ASF consumed[1]	16.30 ± 5.89	16.84 ± 5.50	ns	17.96 ± 7.55	16.44 ± 9.86	ns	16.44 ± 8.53	13.72 ± 9.24	**0.051**
Child Milk-specific indicators	24 h	Consumed milk[2]	97%	100%	ns	93%	79%	**0.01**	84%	67%	**0.007**
		Amount of milk consumed (ml)[1]	306 ± 165	261 ± 121	ns	386 ± 194	323 ± 237	ns	331 ± 219	252 ± 209	**0.02**
		Milk volume sufficient for age?[2]	22%	8%	ns	32%	29%	ns	26%	10%	**0.01**
		Milk ml/kg[1]	25.36 ± 14.39	20.12 ± 10.73	ns	28.11 ± 14.36	27.49 ± 21.00	ns†	22.54 ± 15.79	17.44 ± 16.12	**0.04**
	7 days	# times milk consumed[1]	12.90 ± 5.14	11.53 ± 4.38	ns	12.86 ± 5.84	11..31 ± 7.06	ns	11.6 ± 6.77	9.27 ± 7.14	**0.03**
		Consumed milk ≤7×[2]	4%	0%	ns	9%	24%	**0.02**	19%	35%	**0.01**
Family Diet indicators*	24 h	DDS[1]	4.22 ± 1.04	3.92 ± 1.18	ns	4.55 ± 1.02	4.21 ± 1.21	ns	4.38 ± 1.11	4.25 ± 1.27	ns
		Consumed at least[1] ASF[2]	88%	77%	ns	95%	76%	**0.0005**	91%	71%	**0.0004**
		# ASF consumed[1]	1.39 ± 0.82	1.15 ± 1.06	ns	1.73 ± 0.90	1.23 ± 0.94	**0.0024**	1.68 ± 0.99	1.41 ± 1.25	**ns**
		Consumed milk[2]	82%	54%	**0.02**	90%	61%	**<0.0001**	83%	52%	**<0.0001**

[1] mean ± SD [2] dichotomous variable DDS, diet diversity score # number * items consumed by someone in the household (other than the enrolled child) † only 147 children (of 272 (54%) enrolled at Round 2) had anthropometry needed for calculating this variable.

Table S5. Relation between child and family diet indicators and household milk production (combined cow and buffalo). Child and family diet indicators were assessed in relation to household milk production, classified above or below the median (3.5 L/d). More indicators related to household milk production in Rounds 2 and 3 compared to Round 1.

TIME			>3.5 L/Day OR ⩽3.5 L/Day								
			Round 1			Round 2			Round 3		
			⩽3.5 L/d	>3.5 L/d	*p*	⩽3.5 L/d	>3.5 L/d	*p*	⩽3.5 L/d	>3.5 L/d	*p*
Child Key Diet Indicators	24 h	DDS [1]	4.45 ± 1.05	4.65 ± 1.09	ns	4.49 ± 1.08	4.78 ± 1.01	**0.03**	4.29 ± 1.21	4.51 ± 1.05	ns
		# ASF consumed[1]	1.75 ± 0.87	1.71 ± 0.78	ns	1.64 ± 0.98	2.04 ± 0.90	**0.001**	1.55 ± 1.00	1.83 ± 0.90	**0.02**
		Consumed at least 1 ASF [2]	98%	99%	ns	90%	98%	**0.003**	87%	95%	**0.02**
	7 days	# times ASF consumed [1]	15.52 ± 6.09	16.89 ± 5.65	ns	15.95 ± 8.63	19.25 ± 7.04	**0.001**	14.23 ± 8.85	18.87 ± 7.69	**<0.0001**
Child Milk-specific indicators:	24 h	Consumed milk [2]	96%	97%	ns	81%	98%	**<0.0001**	73%	95%	**<0.0001**
		Amount of milk consumed (ml) [1]	273.3 ± 155.9	325.6 ± 166.2	**0.01**	3240.3 ± 2160.3	4210.4 ± 1790.8	**0.0003**	273.9 ± 217.4	390.5 ± 202.8	**<0.0001**
		Milk volume sufficient for age? [2]	19%	22%	ns	23%	39%	**0.01**	15%	36%	**0.0002**
		Milk ml/kg [1]	21.97 ± 12.8	27.28 ± 14.8	**0.003**	280.3 ± 180.5	270.8 ± 120.8	**ns** †	18.8 ± 16.0	26.2 ± 14.6	**0.0003**
	7 days	# times milk consumed [1]	11.83 ± 5.04	13.52 ± 5.05	**0.009**	110.2 ± 60.63	130.8 ± 50.27	**0.001**	9.77 ± 7.06	13.57 ± 5.86	**<0.0001**
		Consumed milk ≤7× [2]	6%	3%	ns	21%	4%	**<0.0001**	71%	92%	**<0.0001**
Family diet indicators *	24 h	Consumed at least 1 ASF [2]	89%	93%	**0.004**	84%	99%	**<0.0001**	89%	99%	**<0.0001**
		# ASF consumed [1]	1.15 ± 0.76	1.53 ± 0.85	**0.0004**	10.42 ± 0.93	10.85 ± 0.88	**0.0004**	1.47 ± 1.09	1.90 ± 0.90	**0.0015**
		DDS [1]	3.99 ± 0.94	4.54 ± 1.07	**<0.0001**	40.36 ± 10.14	40.61 ± 0.97	**ns**	4.20 ± 1.20	4.61 ± 0.98	**0.006**
		Consumed milk [2]	67%	91%	**<0.0001**	70%	98%	**<0.0001**	64%	99%	**<0.0001**

[1] mean ± SD [2] dichotomous variable DDS, diet diversity score; ASF animal source food; # number

* items consumed by someone in the household (other than the enrolled child); † only 147 children (of 272 (54%) enrolled at Round 2) had the anthropometry needed for calculating this variable.

Table S6. Relation of child and family diet indicators to dairy animal ownership. Mixed effect regressions (logistic and linear) were performed to assess the change in diet indicators over the three survey rounds, after adjusting for dairy animal ownership (none vs >1 adult dairy animal) and child and household characteristics. Significant results related to dairy animal ownership are shown in bold

	Child										*Family*			
	Key Diet Indicators				*Milk-Specific Diet Indicators*						*Diet Indicators*			
	24 h			*1 Week*	*24 h*				*1 Week*		*24 h*			
	DDS	# of ASF consumed	Consumed at least 1 ASF[1]	# of times ASF consumed	Consumed milk [1]	Amount of milk consumed (mL)	Milk volume sufficient for age?[1]	Milk mL/kg	# of times milk consumed	Consumed milk ⩽ 7× [1]	DDS	# of ASF consumed	Consumed at least 1 ASF [1]	Consumed milk [1]
Round Ref = Round 1														
Round 2	0.04 (−0.18, 0.25)	0.1 (−0.08, 0.28)	0.62 (0.37, 1.03)	2.228** (0.92, 3.53)	0.188** (0.07, 0.53)	57.746** (23.35, 92.15)	3.07 *** (1.72, 5.49)	4.051* (0.95, 7.16)	0.39 (−0.67, 1.45)	0.266 *** (0.15, 0.48)	0.18 (−0.03, 0.39)	0.298** (0.12, 0.48)	1.8 (0.87, 3.71)	1.36 (0.74, 2.51)
Round 3	−0.229* (−0.44, −0.02)	−0.07 (−0.24, 0.11)	1.06 (0.66, 1.69)	0.7 −-0.61, 2.01)	0.085 *** (0.03, 0.24)	11.21 (−23.31, 45.73)	1.819 * (1.02, 3.25)	0.38 (−2.33, 3.08)	−0.72 (−1.78, 0.34)	0.162 *** (0.09, 0.29)	0.06 (−0.14, 0.27)	0.318 *** (0.14, 0.49)	1.25 (0.65, 2.39)	0.9 (0.51, 1.59)
Has at least 1 adult cow or buffalo	−0.02 (−0.26, 0.23)	0.11 (−0.1, 0.31)	0.89 (0.51, 1.55)	1.6 (−0.05, 3.24)	**2.877* (1.29, 6.43)**	**58.724 ** (15.44, 102.01)**	**2.314 * (1.08, 4.94)**	**4.659* (0.91, 8.4)**	**1.603 * (0.28, 2.93)**	1.2 (0.64, 2.27)	0.2 (−0.03, 0.44)	**0.347** (0.14, 0.55)**	**4.771 *** (2.56, 8.89)**	**5.807 *** (3.15, 10.7)**
Household wealth	0.165** (0.07, 0.26)	0.14** (0.06, 0.22)	1.347 * (1.06, 1.71)	1.413 *** (0.68, 2.14)	1.59 * (1.08, 2.33)	21.073 * (1.79, 40.35)	1.09 (0.81, 1.47)	0.83 (−0.72, 2.38)	0.865** (0.28, 1.45)	1.09 (0.83, 1.43)	0.248 *** (0.15, 0.34)	0.208 *** (0.13, 0.29)	1.52 ** (1.14, 2.03)	1.567 ** (1.19, 2.06)
Income per capita	0 (0,0)	0 ** (0,0)	1 (1,1)	0 ** (0,0)	1 (1,1)	0 (0,0)	1 (1,1)	0 (0,0)	0 (0,0)	1 (1,1)	0 (0,0)	0 ** (0,0)	1 (1,1)	1 (1,1)
Child age	0 (0,0.01)	0 (0,0.01)	1 (0.99, 1.01)	−0.045* (−0.08, −0.01)	1.024* (1, 1.05)	1.049* (0.02, 2.08)	0.981* (0.97, 1)	−0.155 *** (−0.24, −0.07)	−0.03 (−0.06, 0)	1.041 *** (1.02, 1.06)	0 (−0.01, 0)	0 (−0.01, 0)	1.01 (0.99, 1.02)	1.01 (1, 1.03)
Child gender Ref = female	0.02 (−0.16, 0.19)	0.1 (−0.05, 0.25)	1 (0.68, 1.48)	1.418* (0.06, 2.78)	1.2 (0.59, 2.44)	42.589* (6.61, 78.57)	2.014* (1.16, 3.5)	2.26 (−0.64, 5.17)	1.201 * (0.12, 2.28)	0.8 (0.49, 1.31)	0.07 (−0.1, 0.23)	0.14 (−0.01, 0.29)	1.32 (0.78, 2.22)	1.45 (0.89, 2.38)
Number of children 0-15 yrs in household	−0.03 (−0.12, 0.06)	−0.03 (−0.1, 0.04)	0.91 (0.74, 1.11)	−0.36 (−0.99, 0.27)	0.74 (0.53, 1.04)	−15.02 (−31.75, 1.71)	0.91 (0.69, 1.19)	−1.01 (−2.37, 0.36)	−0.47 (−0.98, 0.04)	0.95 (0.75, 1.21)	0.01 (−0.07, 0.1)	0 (−0.07, 0.07)	1.06 (0.81, 1.39)	0.96 (0.76, 1.22)
Mother's education Ref = No edu														
Some or completed primary school	0.25 (−0.1, 0.6)	0 (−0.3, 0.3)	0.92 (0.39, 2.17)	0.84 (−1.91, 3.59)	1.53 (0.4, 5.82)	39.3 (−33.28, 111.87)	1.56 (0.49, 4.98)	2.35 (−3.55, 8.26)	0.74 (−1.44, 2.93)	2.717* (1.02, 7.22)	0.33 (−0.01, 0.66)	−0.02 (−0.31, 0.28)	2.01 (0.8, 5.08)	1.9 (0.75, 4.81)

Some or completed secondary school	0.09 (−0.27, 0.45)	0.19 (−0.12, 0.49)	1.6 (0.67, 3.79)	0.8 (−2.01, 3.61)	1.29 (0.34, 4.82)	48.89 (−25.31, 123.08)	1.66 (0.51, 5.42)	3.26 (−2.74, 9.27)	−0.09 (−2.32, 2.15)	2.64 (0.98, 7.16)	0.15 (−0.19, 0.49)	0.28 (−0.02, 0.59)	2.01 (0.8, 5.02)	1.79 (0.71, 4.54)
SLC or beyond	0.17 (−0.18, 0.52)	−0.06 (−0.36, 0.24)	1.26 (0.54, 2.95)	−0.64 (−3.4, 2.11)	0.57 (0.16, 2.08)	−24.06 (−96.77, 48.65)	1.06 (0.33, 3.42)	−1.49 (−7.39, 4.41)	−0.74 (−2.93, 1.45)	1.77 (0.68, 4.64)	0.32 (−0.01, 0.66)	0.04 (−0.26, 0.33)	2.62* (1.03, 6.67)	1.5 (0.6, 3.74)

*** < 0.001, ** < 0.01, * < 0.05 [1]Logistic regression DDS Diet diversity score; ASF animal source foods, # number; SLC school leaving certificate; Ref reference; edu education (95%CI).

Table S7. Relation of child and family diet indicators to household milk production (> or ≤3.5 L/d). Mixed effect regressions (logistic and linear) were performed to assess the change in diet indicators over the three survey rounds, after adjusting for household milk production (≤3.5 L/d vs >3.5 L/d, combined cow and buffalo milk) and child and household characteristics. Significant results related to milk production are shown in bold.

	Child										*Family*			
	Key Diet Indicators				*Milk-Specific Indicators*						*Diet Indicators*			
	24 h			*1 week*	*24 h*				*1 week*		*24 h*			
	DDS	# of ASF consumed	Consumed at least 1 ASF[1]	# of times ASF consumed	Consumed milk [1]	Amount of milk consumed (mL)	Milk volume sufficient for age?[1]	Milk mL/kg	# of times milk consumed	Consumed milk ≤7× [1]	DDS	# of ASF consumed	Consumed at least 1 ASF[1]	Consumed milk [1]
Round Ref = Round 1														
Round 2	0.05 (−0.16, 0.26)	0.1 (−0.08, 0.27)	0.63 (0.38, 1.05)	2.202** (0.92, 3.49)	0.156** (0.05, 0.46)	55.991** (22.15, 89.83)	2.948 *** (1.66, 5.22)	3.721* (0.65, 6.8)	0.35 (−0.69, 1.39)	0.264 *** (0.15, 0.47)	0.18 (−0.02, 0.39)	−0.185* (−0.35, −0.02)	1.49 (0.73, 3.04)	1.22 (0.65, 2.29)
Round 3	−0.19 (−0.4, 0.02)	−0.05 (−0.23, 0.12)	1.1 (0.69, 1.77)	0.96 (−0.34, 2.27)	0.084 *** (0.03, 0.25)	18.12 (−16.21, 52.46)	1.946* (1.09, 3.48)	0.66 (−2.03, 3.36)	−0.5 (−1.56, 0.55)	0.165 *** (0.09, 0.3)	0.11 (−0.1, 0.31)	−0.176* (−0.34, −0.02)	1.27 (0.66, 2.42)	1.04 (0.57, 1.88)
Milk production > 3.5 L per day (median)	0.16 (−0.01, 0.33)	0.14 (0, 0.28)	1.12 (0.76, 1.65)	**2.211 *** (1.09, 3.34)**	**7.448 *** (2.97, 18.69)**	**67.982 *** (38.41, 97.55)**	**2.121** (1.31, 3.43)**	**4.331** (1.85, 6.81)**	**1.994 *** (1.09, 2.9)**	1.24 (0.79, 1.96)	**0.313 *** (0.15, 0.48)**	**0.242 *** (0.11, 0.37)**	**6.106 *** (3.06, 12.17)**	**11.876 *** (6.17, 22.88)**
Household wealth	0.149** (0.05, 0.25)	0.133** (0.05, 0.22)	1.323* (1.04, 1.68)	1.297 *** (0.57, 2.02)	1.532* (1.03, 2.27)	18.15 (−0.82, 37.13)	1.07 (0.8, 1.43)	0.61 (−0.94, 2.15)	0.769** (0.19, 1.35)	1.08 (0.82, 1.42)	0.23 *** (0.14, 0.32)	0.142 *** (0.07, 0.22)	1.476** (1.1, 1.98)	1.484** (1.12, 1.96)
Income per capita	0 (0,0)	0* (0,0)	1 (1,1)	0** (0,0)	1 (1,1)	0 (0,0)	1 (1,1)	0 (0,0)	0 (0,0)	1 (1,1)	0 (0,0)	0 (0,0)	1 (1,1)	1 (1,1)
Child age	0 (0,0.01)	0 (0,0.01)	1 (0.99, 1.01)	−0.043* (−0.08, 0)	1.028* (1, 1.05)	1.092* (0.09, 2.1)	0.981* (0.97, 1)	−0.151 *** (−0.23, −0.07)	−0.03 (−0.06, 0.01)	1.041 *** (1.02, 1.06)	0 (−0.01, 0)	0 (0,0)	1.01 (0.99, 1.02)	1.02 (1, 1.03)
Child gender Ref = female	0.01 (−0.17, 0.18)	0.09 (−0.06, 0.24)	0.99 (0.67, 1.47)	1.23 (−0.12, 2.58)	0.94 (0.44, 2.01)	36.781* (1.56, 72)	1.892* (1.1, 3.24)	1.84 (−1.04, 4.71)	1.03 (−0.03, 2.1)	0.79 (0.48, 1.29)	0.04 (−0.13, 0.2)	−0.01 (−0.14, 0.13)	1.16 (0.67, 1.98)	1.2 (0.72, 2.02)

Number of children 0-15 yrs in household	−0.04 (−0.12, 0.05)	−0.03 (−0.11, 0.04)	0.9 (0.74, 1.1)	−0.41 (−1.04, 0.22)	0.673* (0.46, 0.98)	−16.31 (−32.77, 0.16)	0.9 (0.69, 1.17)	−1.02 (−2.37, 0.33)	−0.511* (−1.01, −0.01)	0.95 (0.75, 1.2)	0.01 (−0.07, 0.09)	−0.02 (−0.09, 0.05)	1.05 (0.8, 1.39)	0.92 (0.71, 1.19)
Mother's education Ref = no edu														
Some or completed primary school	0.28 (−0.08, 0.63)	0.01 (−0.29, 0.31)	0.94 (0.4, 2.23)	1.02 (−1.7, 3.74)	1.88 (0.46, 7.7)	44.36 (−26.6, 115.33)	1.66 (0.53, 5.2)	2.89 (−2.96, 8.74)	0.9 (−1.25, 3.05)	2.758* (1.04, 7.34)	0.359* (0.03, .69)	0.04 (−0.23, 0.31)	2.32 (0.88, 6.1)	2.49 (0.92, 6.74)
Some or completed secondary school	0.13 (−0.23, 0.48)	0.2 (−0.1, 0.51)	1.66 (0.7, 3.92)	1.03 (−1.75, 3.81)	1.66 (0.41, 6.75)	55.02 (−17.48, 127.52)	1.78 (0.56, 5.68)	3.66 (−2.28, 9.6)	0.11 (−2.09, 2.3)	2.7 (1, 7.32)	0.19 (−0.15, 0.53)	0.15 (−0.13, 0.42)	2.32 (0.89, 6.06)	2.36 (0.87, 6.36)
SLC or beyond	0.2 (−0.15, 0.55)	−0.05 (−0.35, 0.25)	1.31 (0.56, 3.05)	−0.51 (−3.23, 2.21)	0.61 (0.16, 2.37)	−20.88 (−91.83, 50.07)	1.09 (0.34, 3.45)	−1.31 (−7.13, 4.52)	−0.63 (−2.78, 1.52)	1.79 (0.68, 4.69)	0.352* (0.02, 0.68)	0.05 (−0.23, 0.32)	2.723* (1.04, 7.15)	1.74 (0.66, 4.62)

*** < 0.001, ** < 0.01, * < 0.05 [1] Logistic regression; SLC = school leaving certificate; # number; Ref = reference; edu = education (95%CI).
